# Prognostic value of metabolic activity of the psoas muscle evaluated by preoperative ^18^F-FDG PET-CT in breast cancer: a retrospective cross-sectional study

**DOI:** 10.1186/s12885-021-08886-2

**Published:** 2021-10-27

**Authors:** Keunyoung Kim, In-Joo Kim, Kyoungjune Pak, Taewoo Kang, Young Mi Seol, Young Jin Choi, Hyojeong Kim

**Affiliations:** 1 Department of Nuclear Medicine and Biomedical Research Institute, Pusan National University Hospital, Busan, South Korea; 2 Department of Surgery, Busan Cancer Center, Pusan National University Hospital and Biomedical Research Institute, Pusan National University Hospital, Busan, South Korea; 3 Department of Hematology-Oncology, Pusan National University School of Medicine and Biomedical Research Institute, Pusan National University Hospital, 179 Gudeok-ro, Seo-gu, Busan, 49241 South Korea

**Keywords:** Breast neoplasms, Insulin resistance, Positron emission tomography-computed tomography, Treatment outcomes

## Abstract

**Background:**

This study aimed to evaluate the potential of metabolic activity of the psoas muscle measured by ^18^F-fluorodeoxyglucose positron emission tomography-computed tomography to predict treatment outcomes in patients with resectable breast cancer.

**Methods:**

The medical records of 288 patients who had undergone surgical resection for stages I–III invasive ductal carcinoma of the breast between January 2014 and December 2014 in Pusan National University Hospital were reviewed. The standardized uptake values (SUVs) of the bilateral psoas muscle were normalized using the mean SUV of the liver. SUVRmax was calculated as the ratio of the maximum SUV of the average bilateral psoas muscle to the mean SUV of the liver. SUVRmean was calculated as the ratio of the mean SUV of the bilateral psoas muscle to the mean SUV of the liver.

**Results:**

Univariate analyses identified a higher T stage, higher N stage, estrogen receptor negativity, progesterone receptor negativity, human epidermal growth factor receptor 2 positivity, triple-negative breast cancer, mastectomy (rather than breast-conserving surgery), SUVRmean > 0.464, and SUVRmax > 0.565 as significant adverse factors for disease-free survival (DFS). Multivariate Cox regression analysis revealed that N3 stage (hazard ratio [HR] = 5.347, *P* = 0.031) was an independent factor for recurrence. An SUVRmax > 0.565 (HR = 4.987, *P* = 0.050) seemed to have a correlation with shorter DFS.

**Conclusions:**

A higher SUVRmax of the psoas muscle, which could be a surrogate marker of insulin resistance, showed strong potential as an independent prognostic factor for recurrence in patients with resectable breast cancer.

**Supplementary Information:**

The online version contains supplementary material available at 10.1186/s12885-021-08886-2.

## Background

There is growing recognition that insulin resistance (IR) is correlated with carcinogenesis and poor cancer outcomes [1, 2]. There are several indexes for measuring IR, such as fasting glucose levels, glucose tolerance, HOMA-IR, and hyperinsulinemic-euglycemic clamp. Although the hyperinsulinemic-euglycemic clamp is the gold standard for investigating IR, it is not convenient to use [3].

Skeletal muscle, which is the most important determinant of IR, is responsible for taking up 70–90% of the glucose from the blood in post-prandial healthy humans [4, 5]. ^18^F-fluorodeoxyglucose positron emission tomography-computed tomography (^18^F-FDG PET-CT), which is used for staging and to rule out distant metastases in various cancers, is a molecular imaging tool used to assess tissue glucose utilization. Skeletal muscle glucose utilization estimated by ^18^F-FDG PET-CT has been reported to correlate with the glucose disposal rate during the hyperinsulinemic-euglycemic clamp test [6–9]. Implementing ^18^F-FDG PET-CT could be a good and practical measure to assess IR, compared to the use of serum insulin levels, in patients with cancer. A retrospective study suggested that psoas muscle ^18^F-FDG uptake could be a promising surrogate marker for existing and incipient metabolic derangement [10]; this study utilized the records of ^18^F-FDG PET-CT assessments that were included in routine wellness checkups.

Several studies have revealed that IR measured by various methods other than ^18^F-FDG PET-CT was a prognostic factor of breast cancer [11–13]. The 8th edition of the American Joint Committee on Cancer has incorporated biomarkers such as estrogen receptor (ER), progesterone receptor (PR), and human epidermal growth factor receptor 2 (HER2) into anatomic tumor-node-metastasis staging [14]. This means that the biological factors of breast cancer are as equally important as anatomic staging. We aimed to investigate a new prognostic factor for IR by extracting figures from clinical ^18^F-FDG PET-CT imaging in patients with breast cancer.

## Methods

### Study population

This was a retrospective cross-sectional study that reviewed 375 women with breast cancer who underwent breast-conserving surgical resection for the treatment of invasive breast cancer (invasive ductal carcinoma [IDC]) between January 2014 and December 2014. Participants with pathologies other than IDC, carcinoma in situ, initially metastatic breast cancer, and double primary malignancies were excluded. Patients with thyroid cancer, stage I uterine cancer, and skin malignancies other than malignant melanoma that had been completely resected were included. Finally, 288 patients were enrolled. All patients regularly visited the hospital for follow-up after completion of therapy according to the protocol, which was developed based on results from physical examinations and additional diagnostic imaging studies, including chest/abdomen computed tomography, bone scan, and torso ^18^F-FDG PET-CT. The recurrence of disease was defined as confirmation of local recurrence or distant metastasis.

This retrospective study was approved by the Institutional Review Board (IRB) of the university hospital, which waived the requirement for written consent (IRB 2011–032-097). The study was performed in accordance with the relevant guidelines and regulations.

### Positron emission tomography-computed tomography protocol

All patients were evaluated with ^18^F-FDG PET-CT for preoperative staging. After fasting for at least 6 h, patients were injected with 5.2 MBq of ^18^F-FDG per kilogram of body weight. Serum glucose levels were less than 120 mg/dL before ^18^F-FDG administration. The ^18^F-FDG PET-CT imaging studies were performed 60 min after the intravenous injection of ^18^F-FDG. The PET-CT scanner used was Biograph 40 (Siemens, Knoxville, TN, USA). The emission scan time per bed position was 3 min, and six bed positions were acquired. The PET data were obtained using a high-resolution whole-body scanner with an axial field of view of 21.6 cm. The average axial resolution varied between 2.0 mm full width at half maximum in the center and 2.4 mm at 28 cm. The average total PET-CT examination time was 20 min. Attenuation correction was performed for all patients with iterative reconstruction. The PET-CT images were analyzed in three different planes: transverse, coronal, and sagittal.

### Standardized uptake value measurement

To obtain a maximum value and an average standardized uptake value (SUV) of the psoas muscle and the liver, the region of interest (ROI) was placed as follows: The 1.00 cm^2^sized circular ROI was drawn manually on the axial image of the ^18^F-FDG PET-CT. Bilateral psoas muscle activity was measured using the CT image acquired during ^18^F-FDG PET-CT and circular ROI at the L2 lumbar vertebra. The average SUVs of the bilateral psoas muscle were normalized using the mean SUV of the liver to exclude potential factors affect SUV [15]. The ratio of ROI activity to the activity of the reference region significantly improved the detection of regional changes in metabolism by intra-subject comparison (SUVR). To measure the mean SUV of the liver, an elliptical ROI was placed on the right lobe of the liver, in the middle part, to avoid a mismatch between the CT and PET images and to avoid artifacts due to respiratory motion, and the values were averaged. SUVRmax was calculated as the ratio of the maximum SUV of the bilateral psoas muscle to the mean SUV of the liver. SUVRmean was calculated as the ratio of the mean SUV of the bilateral psoas muscle to the mean SUV of the liver (Formula 1 &2).

1 $$ \mathrm{SUVRmax}=\frac{\raisebox{1ex}{$\left(\mathrm{maximum}\ \mathrm{SUV}\ \mathrm{of}\ \mathrm{right}\ \mathrm{psoas}\ \mathrm{muscle}+\mathrm{maximum}\ \mathrm{SUV}\ \mathrm{of}\ \mathrm{left}\ \mathrm{psoas}\ \mathrm{muscle}\right)$}\!\left/ \!\raisebox{-1ex}{$2$}\right.}{\mathrm{mean}\ \mathrm{SUV}\ \mathrm{of}\ \mathrm{liver}} $$

2 $$ \mathrm{SUVR}\kern0.17em \mathrm{mean}=\frac{\raisebox{1ex}{$\left(\mathrm{mean}\ \mathrm{SUV}\ \mathrm{of}\ \mathrm{right}\ \mathrm{psoas}\ \mathrm{muscle}+\mathrm{mean}\ \mathrm{SUV}\ \mathrm{of}\ \mathrm{left}\ \mathrm{psoas}\ \mathrm{muscle}\right)$}\!\left/ \!\raisebox{-1ex}{$2$}\right.}{\mathrm{mean}\ \mathrm{SUV}\ \mathrm{of}\ \mathrm{liver}} $$

### Statistical analyses

Continuous data are expressed as the median and interquartile range (IQR), and categorical data are presented as the frequency and percentage. For the K-M survival analysis which need binary data, we should find out cutoff value from the continuous variables of SUVRmax and SUVRmean. For the optimal cutoff levels for SUVRmax and SUVRmean for the patients who were survived as disease free or not, a receiver operating characteristic (ROC) analysis was based on the evaluation of the area under the curve (AUC) and 95% confidence interval. The empirical ROC is a plot of the true positive rate versus the false positive rate for all possible cut-off values. In those cut-off values, we could choose one threshold value which has the best performance for the discrimination of the patients who had progressive disease or not. Disease-free survival (DFS) was estimated using the Kaplan–Meier method, and statistical differences were assessed using the log-rank test. A multivariate analysis was performed using the Cox proportional hazards model including variables that had *P*-values < 0.05 in the univariate analyses. The statistical analyses were performed using the Statistical Package for the Social Sciences Statistics for Windows version 22.0 (IBM Corp., Armonk, NY, USA). A *P*-value less than 0.05 was considered as significant.

## Results

### Baseline characteristics

Table 1 shows the baseline characteristics of the 288 enrolled patients with breast cancer. The median follow-up period was 72.5 (IQR, 0.684–0.842) months for all eligible patients. The median age at the time of surgery was 52 (IQR, 44.986–59.629) years. The median value for body mass index (BMI) was 22.803 (IQR, 20.812–25.333) kg/m^2^. Less than 10% of the patients had diabetes mellitus and dyslipidemia. A Chi-squared test or Fisher’s exact test revealed that age, level of BMI, prevalence of diabetes mellitus, rate of menopause, prevalence of dyslipidemia, level of Ki-67, rate of chemotherapy, and rate of radiotherapy were not different between the recurrence and no recurrence groups. Higher T and N stages, ER and PR negativity, HER2 positivity, triple-negative breast cancer (TNBC), mastectomy, high SUVRmean, and high SUVRmax were significantly observed in the recurrence group (Table 1).

**Table 1:** Baseline characteristics

	Recurred (*n* = 26)	Not recurred (*n* = 262)	Total (*n* = 288)	*P*-value
Median age, years (range)	57 (29–76)	52 (29–82)	52 (29–82)	0.490^a^
Follow-up, month (range)	55 (9.5–89.4)	72.8 (0.9–83.1)	72.5 (0.9–89.4)	0.019^a^
BMI (kg/m^2^)	23.1	22.7	22.8	0.704^a^
DM, n (%)				
No	24 (92.3)	238 (90.8)	262 (91.0)	> 0.999^b^
Yes	2 (7.7)	24 (9.2)	26 (9.0)	
Menopause, n (%)				
No	9 (34.6)	114 (43.5)	123 (42.7)	0.382^a^
Yes	17 (65.4)	148 (56.5)	165 (57.3)	
Dyslipidemia, n (%)				
No	25 (96.2)	251 (95.8)	276 (95.8)	> 0.999^b^
Yes	1 (3.8)	11 (4.2)	12 (4.2)	
T stage, n (%)				
T1	5 (19.2)	131 (50.0)	136 (47.2)	< 0.001^a^
T2	13 (50.0)	113 (43.1)	126 (43.8)	
T3	8 (30.8)	17 (6.5)	25 (8.7)	
T4	0	1 (0.4)	1 (0.3)	
N stage, n (%)				
N0	9 (34.6)	167 (63.7)	176 (61.1)	< 0.001^a^
N1	7 (26.9)	72 (27.5)	79 (27.4)	
N2	4 (15.4)	15 (5.7)	19 (6.6)	
N3	6 (23.1)	8 (3.1)	14 (4.9)	
ER, n (%)				
Negative	16 (61.5)	62 (23.7)	78 (27.1)	< 0.001^a^
Positive	10 (38.5)	200 (76.3)	210 (72.9)	
PR, n (%)				
Negative	18 (69.2)	83 (31.7)	101 (35.1)	< 0.001^a^
Positive	8 (30.8)	179 (68.3)	187 (64.9)	
HER2, n (%)				
Negative	11 (42.3)	173 (66.0)	184 (63.9)	0.016^a^
Positive	15 (57.7)	89 (34.0)	104 (36.1)	
Ki-67, n (%)				
< 14%	4 (15.4)	79 (30.2)	83 (28.8)	0.160^a^
≥ 14%	20 (76.9)	182 (69.5)	202 (70.1)	
Not available	2 (7.7)	1 (0.4)	3 (1.0)	
TNBC, n (%)				
No	19 (73.1)	234 (89.3)	253 (87.8)	0.025^b^
Yes	7 (26.9)	28 (10.7)	35 (12.2)	
Surgery, n (%)				
Breast-conserving surgery	10 (38.5)	194 (74.0)	84 (29.2)	< 0.001^a^
Mastectomy	16 (61.5)	68 (26.0)	204 (70.8)	
Chemotherapy, n (%)				
No	2 (7.7)	24 (9.2)	26 (9.0)	> 0.999^b^
Yes	24 (92.3)	238 (90.8)	262 (91.0)	
Adjuvant radiotherapy, n (%)				
No	6 (23.1)	49 (18.7)	55 (19.1)	0.644
Yes	20 (76.9)	205 (78.2)	225 (78.1)	
Lost to follow-up	0	8 (3.1)	8 (2.8)	
SUVmean	0.910 (0.766–1.082)	0.869 (0.792–1.093)	0.904 (0.767–1.087)	0.767
SUVmax	1.006 (0.851–1.177)	0.941 (0.829–1.260)	1.006 (0.845–1.179)	0.956
SUVRmean	0.414 (0.365–0.464)	0.471 (0.382–0.495)	0.417 (0.367–0.469)	0.046
SUVRmax	0.454 (0.400–0.500)	0.488 (0.400–0.575)	0.455 (0.400–0.506)	0.093
SUVRmean, n (%)				
Low	11 (42.3)	195 (74.4)	206 (71.5)	0.001^a^
High	15 (57.7)	67 (25.6)	82 (28.5)	
SUVRmax, n (%)				
Low	18 (69.2)	244 (93.1)	262 (91.0)	0.001^b^
High	8 (30.8)	18 (6.9)	26 (9.0)	

*BMI* body mass index, *DM* diabetes mellitus, *ER* estrogen receptor, *PR* progesterone receptor, *HER2* human epidermal growth factor receptor 2, *TNBC* triple-negative breast cancer, ^a^chi-squared test, ^b^Fisher exact test

### Determination of the cutoff values of SUVRmax and SUVRmean

Based on the ROC curve, the optimal cutoff value for SUVRmax to predict DFS was 0.565, with an AUC of 0.519 (sensitivity 31.6%, specificity 91.3%). The optimal cutoff value for SUVRmean to predict DFS was 0.464, with an AUC of 0.504 (sensitivity 42.1%, specificity 73.9%). Patients were divided into two groups, stratified by the optimal cutoff value of the SUVRmax: 262 patients (90.972%) were assigned to the low SUVRmax group, and 26 patients (9.028%) were assigned to the high SUVRmax group. Using the cutoff value of the SUVRmean, 206 patients (71.528%) were assigned to the low SUVRmean group, and 82 patients (28.472%) were assigned to the high SUVRmean group.

### Prognostic factors for cancer recurrence

**Fig. 1:**
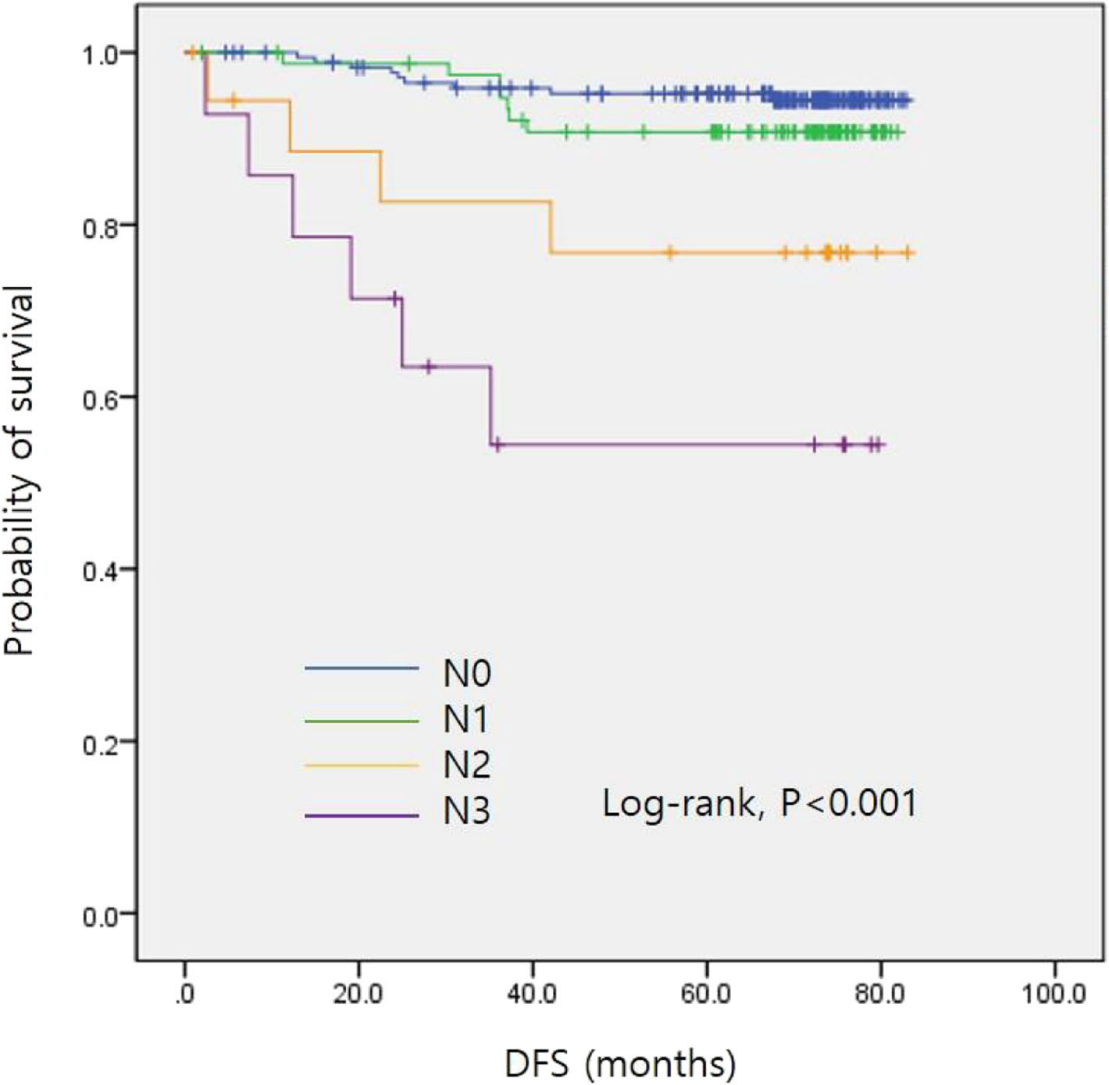
Disease-free survival curves by N stage. DFS, disease-free survival

**Fig. 2:**
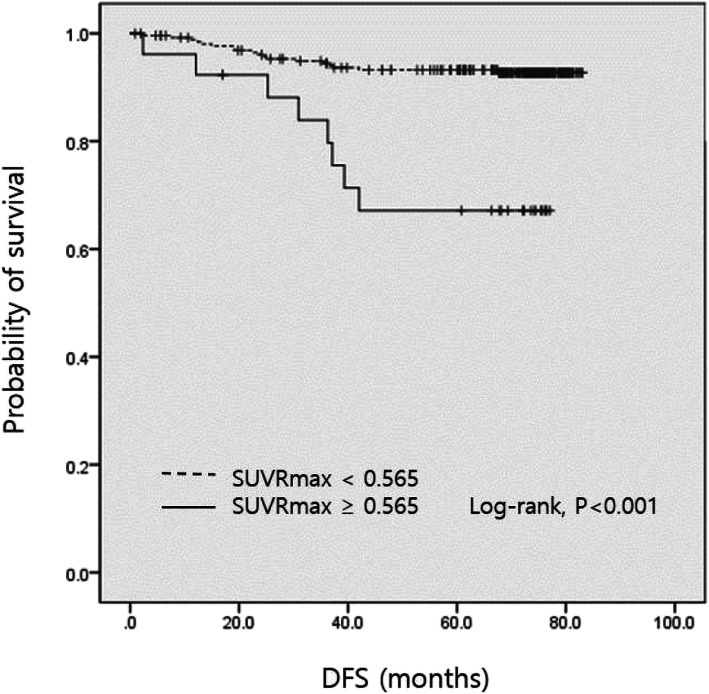
Disease-free survival curves by SUVRmax values. DFS, disease-free survival; SUVRmax, ratio of the maximum standard uptake value

The univariate analyses identified a higher T stage, higher N stage, ER negativity, PR negativity, HER2 positivity, TNBC, mastectomy (rather than breast-conserving surgery), SUVRmean > 0.464, and SUVRmax > 0.565 as significant factors that predicted disease recurrence after initial disease management (Table 2). The final multivariate Cox regression analysis showed that N3 stage (hazard ratio [HR] = 5.347, *P* = 0.031) was an independent factor for recurrence (Table 2). The DFS curves according to N stages are shown in Fig. 1. Moreover, increased SUVRmax (HR = 4.987, *P* = 0.050) showed strong potential as an independent prognostic factor for cancer recurrence. Figure 2 shows division of the DFS curves based on the level of SUVRmax.

**Table 2:** Cox proportional hazards model for disease recurrence

Covariate	Univariate analysis		Multivariate analysis	
	HR (95% CI)	*P*-value	HR (95% CI)	*P*-value
T stage				
T2	2.89 (1.030–8.106)	0.044	1.645 (0.548–4.935)	0.374
T3	11.638 (3.801–35.633)	< 0.001	2.658 (0.551–12.819)	0.223
N stage				
N1	1.716 (0.639–4.608)	0.284	1.267 (0.413–3.889)	0.679
N2	4.938 (1.521–16.037)	0.008	1.749 (0.318–9.631)	0.521
N3	12.961 (4.582–36.666)	< 0.001	5.347 (1.162–24.603)	0.031
ER	0.206 (0.093–0.453)	< 0.001	0.873 (0.228–3.348)	0.843
PR	0.212 (0.092–0.487)	< 0.001	0.783 (0.194–3.163)	0.731
HER2	2.592 (1.190–5.643)	0.016	2.843 (0.743–10.872)	0.127
TNBC	2.963 (1.245–7.052)	0.014	4.505 (0.801–25.329)	0.088
Breast-conserving surgery	0.224 (0.102–0.495)	< 0.001	0.502 (0.187–1.352)	0.173
SUVRmean	3.633 (1.668–7.911)	0.001	2.007 (0.755–5.335)	0.163
SUVRmax	4.987 (2.167–11.475)	< 0.001	3.014 (1.002–9.071)	0.050

*ER* estrogen receptor, *PR* progesterone receptor, *HER2* human epidermal growth factor receptor 2, *TNBC* triple-negative breast cancer, SUVRmax, ratio of the maximum standard uptake value; SUVRmean, ratio of the mean standard uptake value

## Discussion

This study showed that increased SUVRmax of the psoas muscle is correlated with shorter DFS in patients with breast cancer. The multivariate analysis with covariates of the TN stage, ER/PR/HER2 status, surgical protocol, SUVRmean, and SUVRmax revealed that N3 stage was an independent prognostic factor. Mastectomy seemed to be correlated with recurrence (Table 1); however, adjustment for the TN stage covariate corrected for the deviation in surgical protocol, which could be attributed to staging (Table 2). The stage and hormone receptor status of breast cancer are considered prognostic factors. Although BMI showed a positive correlation with SUVRmax, it was not an independent prognostic factor in the multivariate analysis.

A retrospective study by Korean researchers demonstrated that higher ^18^F-FDG uptake (SUVmax) in the psoas muscle was positively correlated with incipient metabolic syndrome (HR = 3.26, *P* = 0.0174) [10]. The study showed that higher SUVmax of the psoas muscle was an independent risk factor for metabolic syndrome among 157 participants with a balanced sex ratio (60% male and 40% female). This result is contradictory with those of previous studies that showed lower uptake in skeletal muscle in a group of patients with IR. According to previous studies that used the gold standard hyperinsulinemic-euglycemic clamp technique [6–9], excess insulin in patients with IR might result in the saturation of overexpressed GLUT4 in the plasma membrane, and the decreased intracellular GLUT4 levels could not respond to infused insulin. This might hinder the uptake of ^18^F-FDG in hyperinsulinemic-euglycemic patients with IR. The ^18^F-FDG PET-CT approach utilized by these Korean researchers was based on a routine clinical setting; thus, it was performed after 4–6 h of fasting. Moreover, the imaging approach used in the present study was similar to that used by the Korean researchers, but this study involved patients with breast cancer.

Another retrospective study comprising 59 patients with esophageal cancer in the United States reported that a higher psoas SUVmax was a favorable prognostic factor for overall survival (HR = 0.37, *P* = 0.04) [16]. They stated that less fatty infiltration of the muscle might result in higher ^18^F-FDG uptake and reflect a more robust muscle tissue in patients. They included 90% of male patients, and 30% of the patients had stage IV esophageal cancer. The small sample size, biased sex ratio, and advanced stage of cancer might have influenced the result. Moreover, they used SUVmax, and we used SUVRmax.

They included 32% of diabetes mellitus (DM) patients while this study has 9% of DM. Although it is not usual that a study regarding IR includes DM, we regard that diagnosed DM patients are well treated. The patients with uncontrolled DM and high level of serum glucose could not take ^18^F-FDG PET-CT in our institution. There are diabetes medications which reduce IR including biaguanides and thiazolidinediones. We consider the level of IR itself which was measured by ^18^F-FDG PET-CT at the time of diagnosis of breast cancer. Differences in SUV values between DM or non-DM patients and correlations between SUV values, FBS and BMI are presented as supplemental tables.

In the context of various cancers, IR has been reported using the hyperinsulinemic-euglycemic clamp technique [17–20]. Several researchers using the same technique have reported that insulin sensitivity had been restored after surgical tumor resection [21, 22]. This suggests that IR in patients with cancer could be caused by the tumor itself. IR accompanied by cancer could be a prognostic factor or surrogate marker for residual disease.

This study has several limitations. This was a retrospective study from a single institution. The study’s small sample size limited subgroup analyses, although breast cancer has various subgroups that have been well established. Additive information such as HOMA-IR data, which could validate high SUVRmax of the psoas muscle as a definite surrogate marker of IR, was not available in this study.

## Conclusion

In conclusion, an increased SUVRmax of the psoas muscle in patients with operable breast cancer could be an unfavorable prognostic factor. Further studies are needed to confirm the utility and reliability of this index for IR in patients with cancer and its role in cancer prognosis.

## Supplementary Information

**Additional file 1: Supplemental Table 1.** Differences in SUV values between DM or non-DM patients. **Supplemental Table 2.** Correlations between SUV values, FBS and BMI.

## Data Availability

The datasets used and/or analysed during the current study are available from the corresponding author on reasonable request.

## Abbreviations

IR: Insulin resistance
HOMA-IR: Homeostasis model assessment of insulin resistance
^18^F-FDG PET-CT: ^18^F-fluorodeoxyglucose positron emission tomography-computed tomography
ER: Estrogen receptor
PR: Progesterone receptor
HER2: Human epidermal growth factor receptor 2
IDC: Invasive ductal carcinoma
IRB: Institutional Review Board
SUV: Standardized uptake value
ROI: Region of interest
IQR: Interquartile range
ROC: Receiver operating characteristic
AUC: Area under the curve
DFS: Disease-free survivals
BMI: Body mass index
TNBC: Triple-negative breast cancer
HR: Hazard ratio
DFS: Disease-free survival
