# Supplementary material for: Prognostic value of metabolic activity of the psoas muscle evaluated by preoperative 18F-FDG PET-CT in breast cancer: a retrospective cross-sectional study
Source: BMC Cancer. 2021 Oct 27;21:1151. doi: 10.1186/s12885-021-08886-2 (PMC8555075; doi:10.1186/s12885-021-08886-2)
Supplement: Supplementary file 1 — Additional file 1: Supplemental Table 1. Differences in SUV values between DM or non-DM patients. Supplemental Table 2. Correlations between SUV values, FBS and BMI. [file 12885_2021_8886_MOESM1_ESM.docx]

Supplemental Table 1 Differences in SUV values between DM or non-DM patients

|  | DM  (n=26) | Non-DM  (n=262) | *P* - value |
| --- | --- | --- | --- |
| SUVmean | 0.891 (0.786–1.104) | 0.904(0.762–1.084) | 0.974 |
| SUVmax | 1.005 (0.912–1.111) | 1.006(0.839–1.183) | 0.853 |
| SUVRmean | 0.388(0.637 – 0.468) | 0.416 (0.367 – 0.469) | 0.572 |
| SUVRmax | 0.466(0.413–0.549) | 0.454 (0.400–0.500) | 0.392 |

Supplemental Table 2 Correlations between SUV values, FBS and BMI

|  | | FBS | BMI |
| --- | --- | --- | --- |
| SUVave | Correlation coefficient Significance Level P | 0.092 0.1450 | 0.218 < 0.001 |
| SUVmax | Correlation coefficient Significance Level P | 0.097 0.1255 | 0.180 0.002 |
| SUVRave | Correlation coefficient Significance Level P | -0.154 0.0144 | -0.000 0.997 |
| SUVRmax | Correlation coefficient Significance Level P | -0.152 0.0158 | -0.058 0.325 |
